# Short-course intravenous beta-lactams for uncomplicated cystitis in hospitalized patients

**DOI:** 10.1017/ash.2025.10101

**Published:** 2025-08-22

**Authors:** Payton Simpson, Katie Wallace, Katherine Olney, Danielle Casaus, David S. Burgess, Aric Schadler, Abigail Leonhard, Jeremy VanHoose

**Affiliations:** 1 Department of Pharmacy Services, University of Kentucky HealthCare, Lexington, KY, USA; 2 Department of Pharmacy Practice and Science, College of Pharmacy, University of Kentucky, Lexington, KY, USA; 3 University of Kentucky Pharmacy and University of Kentucky HealthCare Pediatrics, Lexington, KY, USA; 4 Dr. Bing Zhang Department of Statistics, College of Arts and Science, University of Kentucky, Lexington, KY, USA

## Abstract

**Purpose:**

Currently, the Infectious Diseases Society of America (IDSA) Guidelines for Uncomplicated Urinary Tract Infections (UTIs) recommend a 3 to 7-day antibiotic course of oral beta-lactam agents when other recommended agents are not feasible. In recent years, studies have demonstrated efficacy in shorter courses of antimicrobial therapy for acute uncomplicated cystitis compared with longer courses, but there is limited data regarding intravenous beta-lactams for acute uncomplicated cystitis.

**Methods:**

This single-center, retrospective, non-inferiority cohort study included adult patients admitted to University of Kentucky Albert B. Chandler Medical Center or Good Samaritan Hospital with acute uncomplicated cystitis. The primary outcome assessed was treatment failure, defined as the need for retreatment with additional antibiotic therapy within 30 days of antibiotic completion. Secondary outcomes include incidence of C. difficile infection within 30 days of antibiotic therapy, hospital readmission, and outpatient telephone encounters within 30 days of discharge. Patients were divided into the short course (those receiving three days or less of beta-lactam antibiotics and at least 1 day was IV) or the long course (those receiving four or more days of beta lactam antibiotics).

**Results:**

Overall, 52 patients met the criteria to be included in the final study, with 33 in the short course beta-lactam group and 19 in the long-course beta-lactam group. Failure rates between short and long course were 15.2% and 15.8% respectively (p=1.000). Ceftriaxone was the most commonly utilized antibiotic in both groups. The median total antibiotic duration between the long and short groups was 3 and 6 days respectively (p<0.001).

**Conclusions:**

In hospitalized patients warranting initial IV therapy for acute uncomplicated cystitis, a 3-day total of beta-lactam therapy, with transition to oral, should be considered.

## Background

Currently, the Infectious Diseases Society of America (IDSA) Guidelines for Uncomplicated Urinary Tract Infections (uUTI) recommend a 3- to 7-day therapy course for oral beta-lactam agents.^1^ Data regarding optimal treatment duration remains unclear given the potential for reduced efficacy with shorter courses of oral beta-lactams. In recent years, studies have demonstrated the efficacy of antibiotic courses of 3 days for uncomplicated cystitis compared with that of longer courses.^2,3^ Prolonged courses of antibiotic therapy contribute to increasing widespread antimicrobial resistance and increased risk for complications such as *C. difficile* infections (CDIs).^1,4^ In both the inpatient and outpatient setting, a 3-day course of antimicrobial therapy with a fluoroquinolone or trimethoprim-sulfamethoxazole (TMP-SMX) is frequently recommended for acute uncomplicated cystitis. Agents such as beta-lactams and nitrofurantoin are typically given for a course of at least 5 days given limited data supporting shorter courses.^1^ Although the majority of patients with uncomplicated cystitis do not require admission to the hospital, patients with acute uncomplicated cystitis warranting hospital admission for a separate indication may initially receive intravenous (IV) antibiotics. Beta-lactam antibiotics are generally well-tolerated by patients and attain favorable urinary drug concentrations. One study examined shorter durations of IV beta-lactams, specifically ceftriaxone, for uncomplicated cystitis.^5^ These data demonstrated non-inferiority when a 3-day course was compared to longer durations, supporting a 3-day course of ceftriaxone for uncomplicated cystitis in the inpatient setting. Our study aimed to compare treatment failure rates of acute uncomplicated cystitis in hospitalized patients treated with short courses of beta-lactams compared with longer courses of beta-lactams.

## Methods

This was a single-center, retrospective, non-inferiority cohort study of adult patients with uUTI at a large academic hospital. Patients had to endorse at least one of the following symptoms: dysuria, urinary frequency, urinary urgency, or altered mental status (AMS) without definitive clinical explanation as described in the providers’ notes during the included hospital encounter. Patients in the short course cohort either completed their entire cystitis treatment with an IV beta-lactam antibiotic or received at least one day of an IV beta-lactam before changing to an oral beta-lactam to complete their therapy course. If a patient was switched to an oral beta-lactam, the total duration of therapy between IV and oral must have been 3 days or less for inclusion into the short course cohort. The long course cohort received more than 3 days of IV and/or oral beta-lactam. Patients were excluded from the study if they had asymptomatic bacteriuria, complicated cystitis, baseline incontinence or urinary symptoms, multiple sources of infection, or identified resistance to empiric antibiotic. Patients on antibiotic therapy initiated in the outpatient setting or outside institution prior to hospital admission, and those who were transitioned to a non-beta-lactam therapy as part of their treatment course were also excluded. International Classification of Diseases 10, (ICD-10) codes for “acute cystitis” and “cystitis, unspecified” were utilized to extract patients from the electronic medical record. Manual chart review assessed urinary symptoms within the provider notes to determine inclusion or exclusion in the study, determination of antibiotic treatment course, and initial treatment susceptibilities. Outpatient records were reviewed for prescription fill history to assess for additional antibiotics after completion of the initial therapy course. IBM SPSS Statistics version 28 was utilized for the purposes of statistical analysis. Categorical variables were analyzed using Pearson’s χ^2^ or Fischer’s exact test as appropriate and are reported with frequencies and percentages. Continuous variables were analyzed utilizing a Mann-Whitney U test and are reported with medians and interquartile ranges (IQR). The primary outcome of this study was the difference in treatment failure rates between the two therapy groups. Treatment failure was defined as the need for retreatment with additional antibiotic therapy within 30 days of initial antibiotic completion. Secondary outcomes included the incidence of CDI, defined as a positive PCR toxin and positive toxin test, within 30 days of antibiotic treatment. Additional secondary outcomes included hospital readmission within 30 days of discharge, and telephone encounters for any reason within 30 days of discharge.

## Results

Fifty-two patients were included in the final analysis with a total of 33 patients in the short course and 19 in the long course. Baseline patient demographics were similar between the two groups (Table 1). The primary outcome of treatment failure rates was similar between the two groups (Table 2). The short-course group had a failure rate of 15.2%, while the long-course was 15.8% (*P* = 1.000). For secondary outcomes, there was no incidence of CDI in either study group. Readmission rates between the short and long course group were 3.0% versus 31.6% respectively (*P* = .007). No patient had a hospital readmission related to a uUTI or urinary symptoms. Median IV beta-lactam duration was 3 days in both the short and long course groups respectively. Median total antibiotic duration was 3 days in the short course group compared with 6 days in the long course group (*P* < .001). Patients in the in the short-course group were significantly less likely to be prescribed antibiotics at discharge when compared to the long course group (6.1% vs 47.7%; *P* < .001).

**Table 1.** Baseline patient demographics

Demographic	Short-course group (n = 33)	Long-course group (n = 19)	*p*-value
**Female—n (%)**	33 (100%)	19 (100%)	
**Age (years) – median (IQR)**	76 (69–82)	73 (63–78)	.299

**Race—n (%)**			
** White**	26 (78.8%)	16 (84.2%)	
** Black/African American**	5 (15.2%)	2 (10.5%)	
** Other/Unknown**	2 (3.7%)	1 (5.3%)	

**BMI kg/m** ^**2**^ **– median (IQR)**	25.79 (22.94–31.23)	31.97 (25.23–37.79)	**.049**
**Maximum WBC – median (IQR)**	7.18 (5.50–9.94)	8.27 (5.85–9.33)	.470
**Max temperature (degrees celsius) – median (IQR)**	36.9 (36.8–37.1)	37.0 (36.8–37.4)	.710
**Serum creatinine on admission (mg/dl) – median (IQR)**	1.02 (.82–1.58)	1.25 (.82–1.65)	.857
**Charlson Co-morbidity Index (CCI) – median (IQR)**	7 (4–9.50)	7 (5–10)	.334
**Diagnosis of immunocompromised—n (%)**	2 (6.1%)	2 (10.5%)	.617
**History of UTI—n (%)**	5 (15.2%)	3 (15.8%)	1.000

**Table 2.** Treatment outcomes

Outcome	Short-course group (n = 33)	Long-course group (n = 19)	*p*-value
**Treatment failure—n (%)**	5 (15.2%)	3 (15.8%)	1.000

**C. Difficile infection within 30 days—n (%)**	0 (0%)	0 (0%)	
**Hospital readmission within 30 days—n (%)**	1 (3.0%)	6 (31.6%)	.007
**UTI-related readmission—n (%)**	0 (0%)	0 (0%)	
**Telephone encounters within 30 days of discharge—n (%)**	13 (39.4%)	9 (47.4%)	.575

**Duration of IV beta-lactam treatment (days) – median (IQR)**	3 (3–3)	3 (2–4)	.312
**Total antibiotic duration (days) – median (IQR)**	3 (3–3)	6 (5–7)	< .001
**Discharge antibiotics prescribed—n (%)**	2 (6.1%)	9 (47.4%)	< .001
**Hospital length of stay (days) – median (IQR)**	3.66 (2.42–8.32)	3.94 (2.92–6.66)	.932

## Discussion

This study aimed to determine if shorter courses (≤3 d) of IV beta-lactam-containing regimens for acute uncomplicated cystitis resulted in similar rates of treatment failure when compared to longer courses (>3 d). Similar to prior studies, we noted that shorter antibiotic courses resulted in similar incidence of treatment failure when compared to longer courses.

There are several strengths to our study. We excluded patients who were on antibiotics at the time of hospital admission. Exclusion of these patients removed potential confounders and allowed for more accurate analysis of total antibiotic duration. Additionally, patients who were transitioned to a non-beta lactam therapy after receiving empiric beta-lactam therapy were excluded to adequately assess for treatment failure rates specifically for beta-lactam monotherapy. Similar failure rates were noted between both groups in our study, with slightly lower, overall rates of treatment success in both groups when compared to prior.^5,6,7^

Limitations to our study include the retrospective nature and relatively small sample size. The small sample size could be attributed to uUTI commonly being managed as an outpatient complication that typically does not require hospitalization, and the strict exclusion criteria utilized in this study. Another limitation is the inclusion of patients with AMS as the only sign of a urinary tract infection. Although AMS has historically been associated with urinary tract infections in older adults, recent studies have suggested a weak association between AMS and presence of a urinary tract infection.^8^ Patients within this study were included only if their AMS resolved as a result of antibiotic treatment to limit confounding factors although we recognize that AMS may have resolved without antibiotics. This study also excluded patients of male gender; however, the forthcoming IDSA UTI guidelines may include male patients in their definition of uncomplicated UTI.^9^ Additionally, recent literature suggests that certain male patients may qualify for shorter courses of antibiotic therapy.^10^

Our study demonstrated that a 3-day course of IV containing beta-lactam therapy may be reasonable for treatment of patients hospitalized with acute uncomplicated cystitis. Further randomized studies with a large study cohort are needed to optimize utilization of IV beta-lactam containing therapy for uUTI in the inpatient setting.
